# Supplementary figures and images for: c-FLIP facilitates ZIKV infection by mediating caspase-8/3-dependent apoptosis
Source: PLoS Pathog. 2024 Jul 22;20(7):e1012408. doi: 10.1371/journal.ppat.1012408 (PMC11293698; doi:10.1371/journal.ppat.1012408)

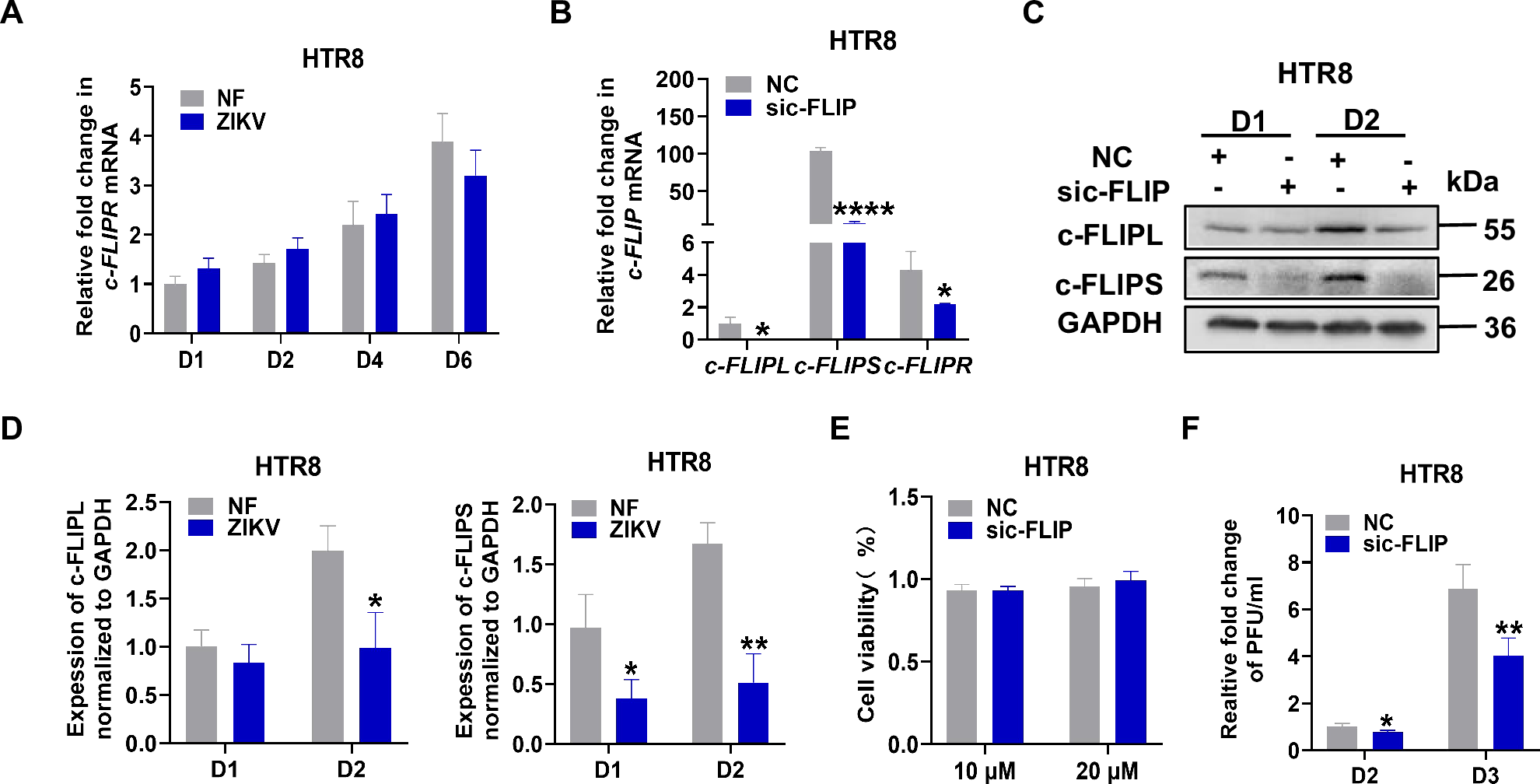

Supplement: S1 Fig — (A) HTR8 cells were infected with ZIKV at a MOI of 1. c-FLIPR levels were measured on D1, D2, D4 and D6 post-infection by qPCR. (B) c-FLIPL, c-FLIPS and c-FLIPR were measured by qPCR post sic-FLIP transfection on day 1. (C) Western blot of c-FLIPL and c-FLIPS expression in HTR8 cells post sic-FLIP transfection on day 1 and day 2 were assessed. (D) Quantification of c-FLIPL and c-FLIPS protein levels relatives to GAPDH. (E) HTR8 cells were transfected with various concentrations of negative control (NC) or sic-FLIP for 1 day, followed by the detection of cell viability using the CCK8 assay. (F) The fold change in Fig 1F. The data are one representative of three independent experiments and analyzed by unpaired Student’s t test. Data are presented as means ± SD. *P <0.05, **P <0.01, ****P < 0.0001 compared to control group. (TIF) [file ppat.1012408.s001.tif]

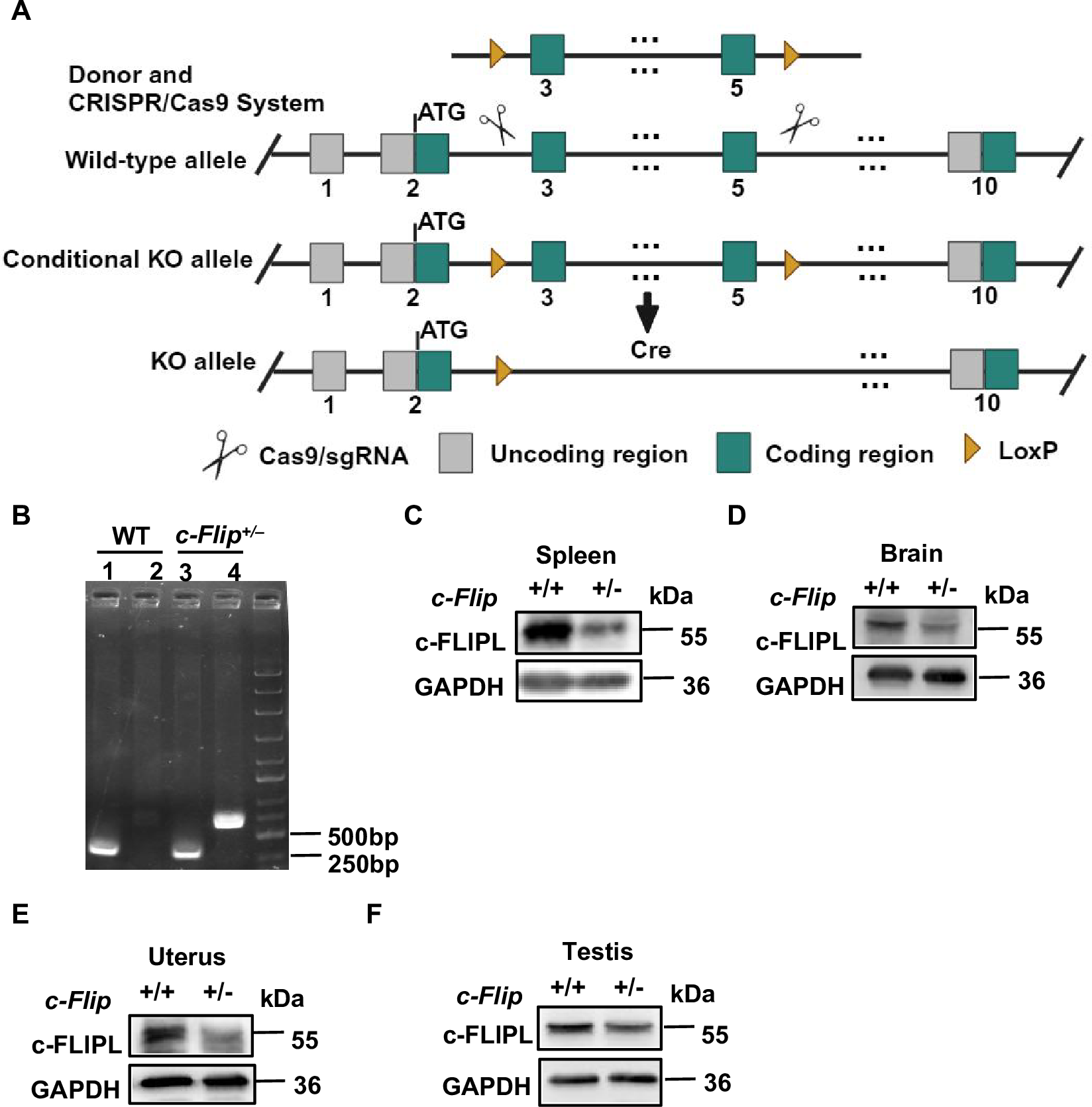

Supplement: S2 Fig — (A) The c-Flip+/- mice were generated by using CRISPR/Cas9 to knockout exon 3–5 of c-FLIP gene. (B) PCR analysis of genomic DNA confirmed the genotypes of WT and c-Flip+/- mice. The first and second lanes depict gene fragments of the c-FLIP chromosome in WT mice, with sizes of 316bp and 6621bp, respectively. The third and fourth lanes show gene fragments of c-FLIP chromosome in c-Flip+/- mice, with sizes of 316bp and 648bp, respectively. (C-F) Western blot assays of c-FLIPL expression in spleen (C), brain (D), uterus (E), and testis (F) of the WT and c-Flip+/- mice. S2A Fig was created with BioRender.com. (TIF) [file ppat.1012408.s002.tif]

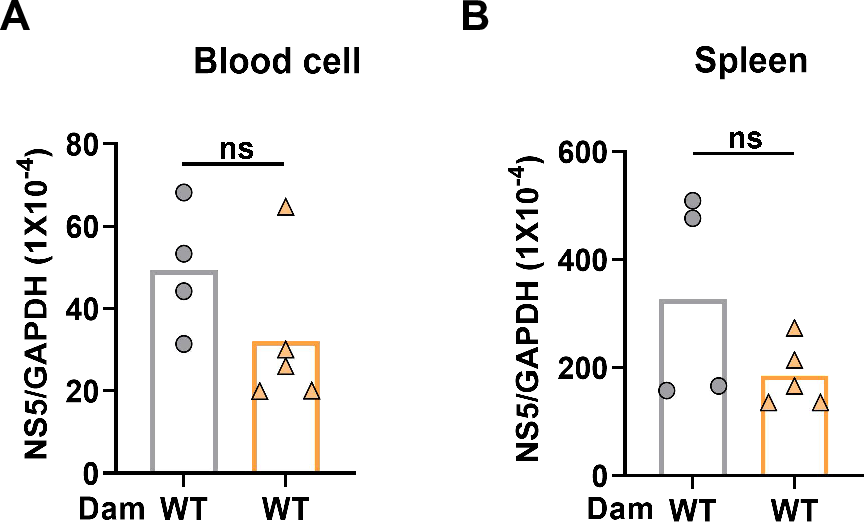

Supplement: S3 Fig — (A-B) ZIKV replication in the blood cell (A) and spleen (B) collected from 4–5 pregnant dams per group was measured by qPCR. Data are analyzed by unpaired Student’s t test and presented as means ± SD. The data represent the collective results of three independent experiments. All the data are analyzed by unpaired Student’s t test. Data are presented as means ± SD. ns indicates a non-significant difference. (TIF) [file ppat.1012408.s003.tif]

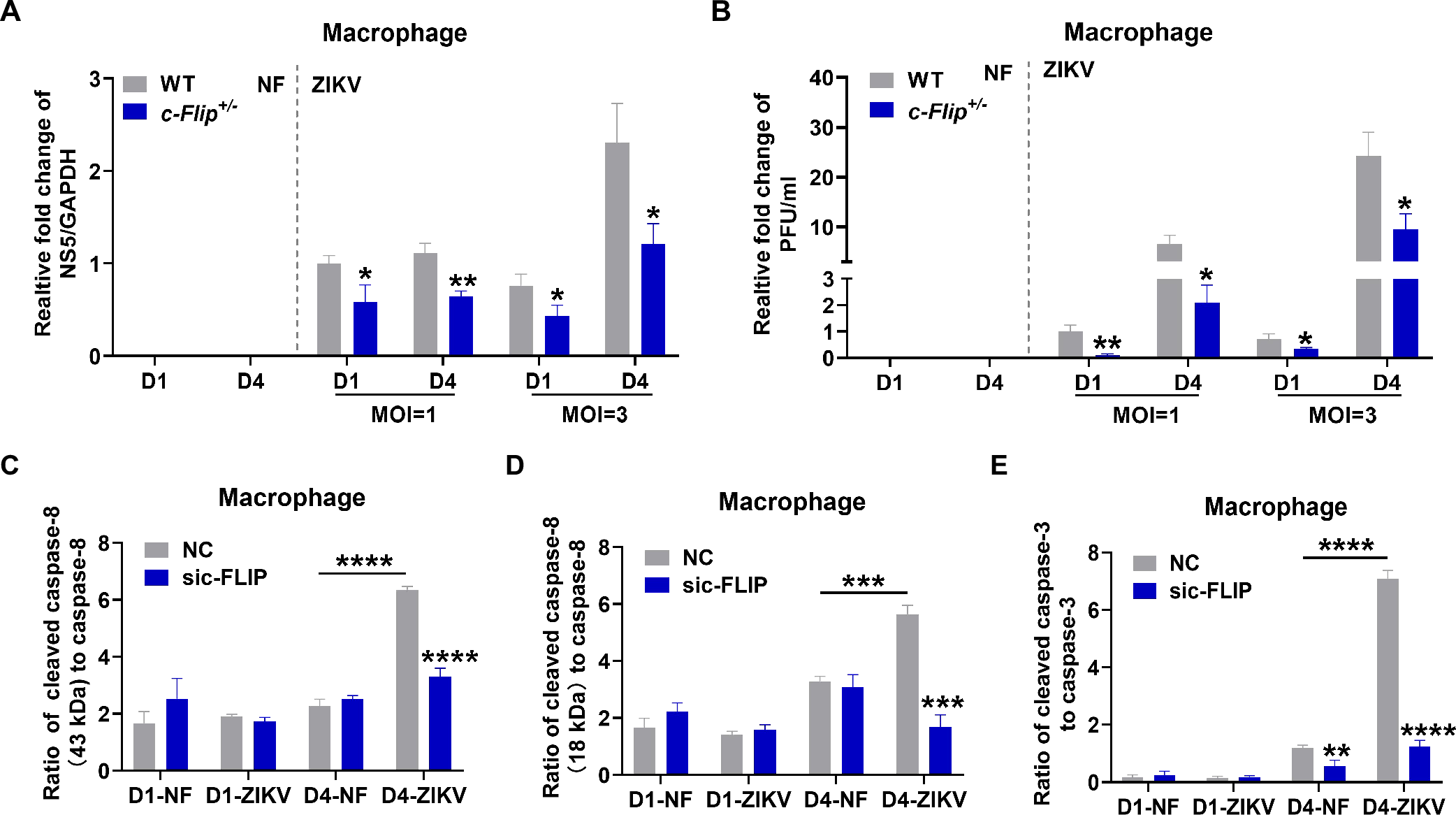

Supplement: S4 Fig — (A-B) The fold change analysis in Fig 6A and 6B. (C-E) Quantification of the western blot in Fig 6D was conducted. The ratio of cleaved caspase-8/3 to total caspase-8/3 was measured by comparing the protein bands between cleaved caspase-8 and total caspase-3 corresponding to each time point. The data represent either a single experiment chosen as representative from three independent experiments (A-B) or the collective results of three independent experiments (C-E). All the data are analyzed by unpaired Student’s t test. Data are presented as means ± SD. *P <0.05, **P < 0.01, ***P < 0.001, ****P < 0.0001 compared to control group. (TIF) [file ppat.1012408.s004.tif]

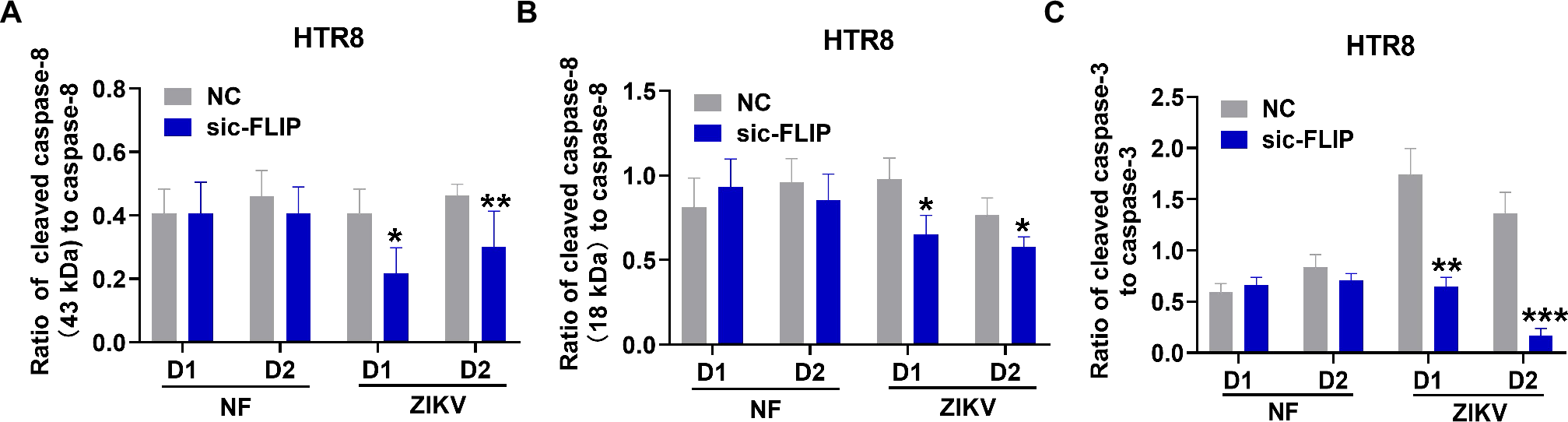

Supplement: S5 Fig — (A-C) Quantification of the western blot in Fig 7C was conducted. The ratio of cleaved caspase-8/3 to total caspase-8/3 was measured by comparing the protein bands between cleaved caspase-8 and total caspase-3 corresponding to each time point. The data represent the collective results of three independent experiments. All the data are analyzed by unpaired Student’s t test. Data are presented as means ± SD. *P <0.05, **P < 0.01, ***P < 0.001 compared to control group. (TIF) [file ppat.1012408.s005.tif]

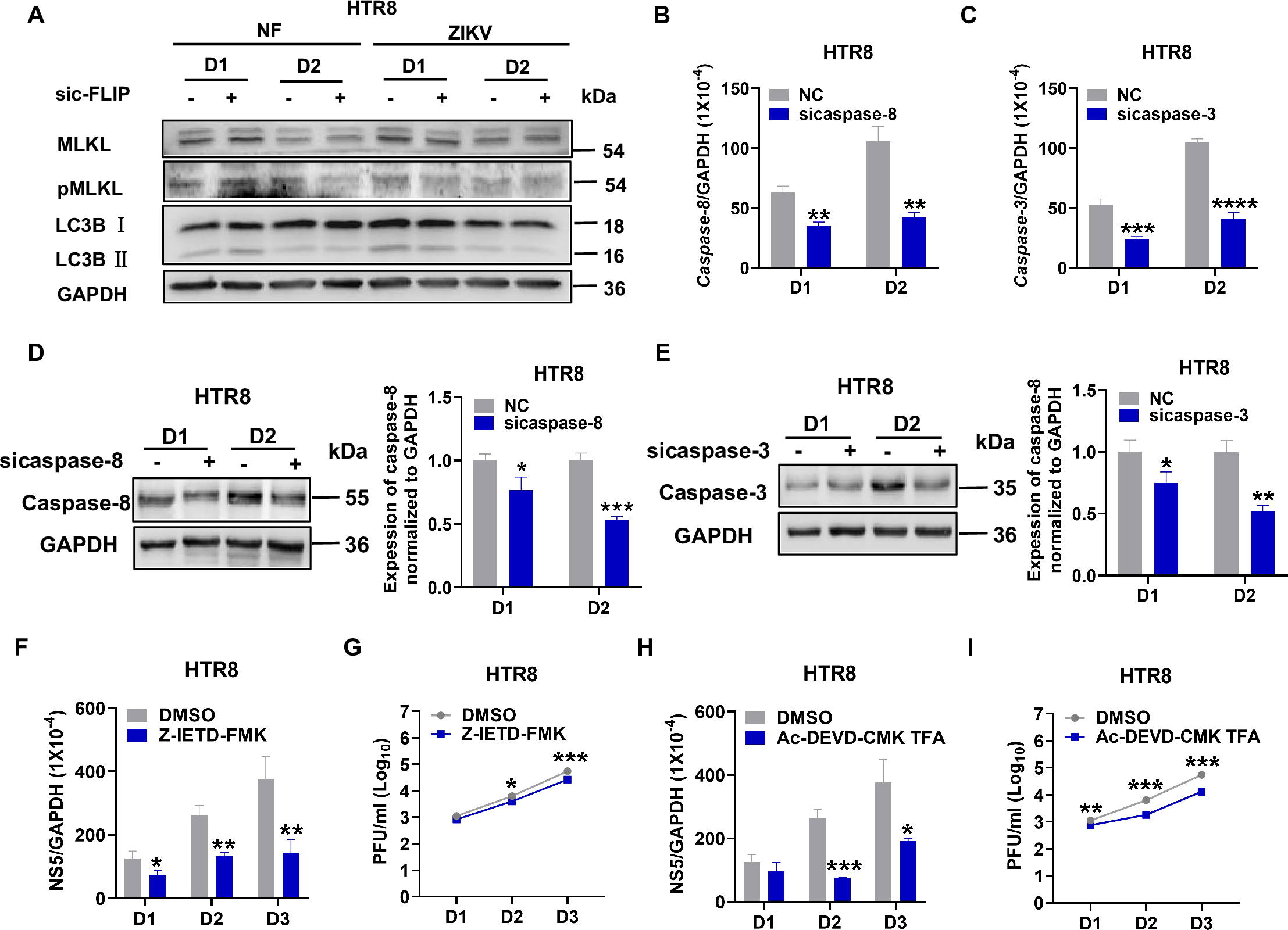

Supplement: S6 Fig — (A) HTR8 cells were infected with ZIKV at a MOI of 1 post sic-FLIP transfection. On D1 and D2 post-infection, MLKL, pMLKL and LC3B levels were measured by western blot. (B-C) Caspase-8 (B) and caspase-3 (C) were measured by qPCR post sicaspase-8 or sicaspase-3 transfection on day 1. (D-E) Western blot assays of caspase-8 (D) or caspase-3 (E) expression in HTR8 cells post sicaspase-8 or sicaspase-3 transfection for 1 day and 2 days. (F-I) HTR8 cells were infected with ZIKV at a MOI of 1 post sicaspase-8 (F-G) or sicaspase-3 (H-I) transfection for 24 hours. The viral load was measured on D1, D2 and D3 post-infection by qPCR (F, H) and plaque assay (H, I). The data represent either a single experiment chosen as representative from three independent experiments (B-C, F-I) or the collective results of three independent experiments (D-E). All the data are analyzed by unpaired Student’s t test. Data are presented as means ± SD. *P <0.05, **P < 0.01, ***P < 0.001, ****P < 0.0001 compared to control group. (TIF) [file ppat.1012408.s006.tif]

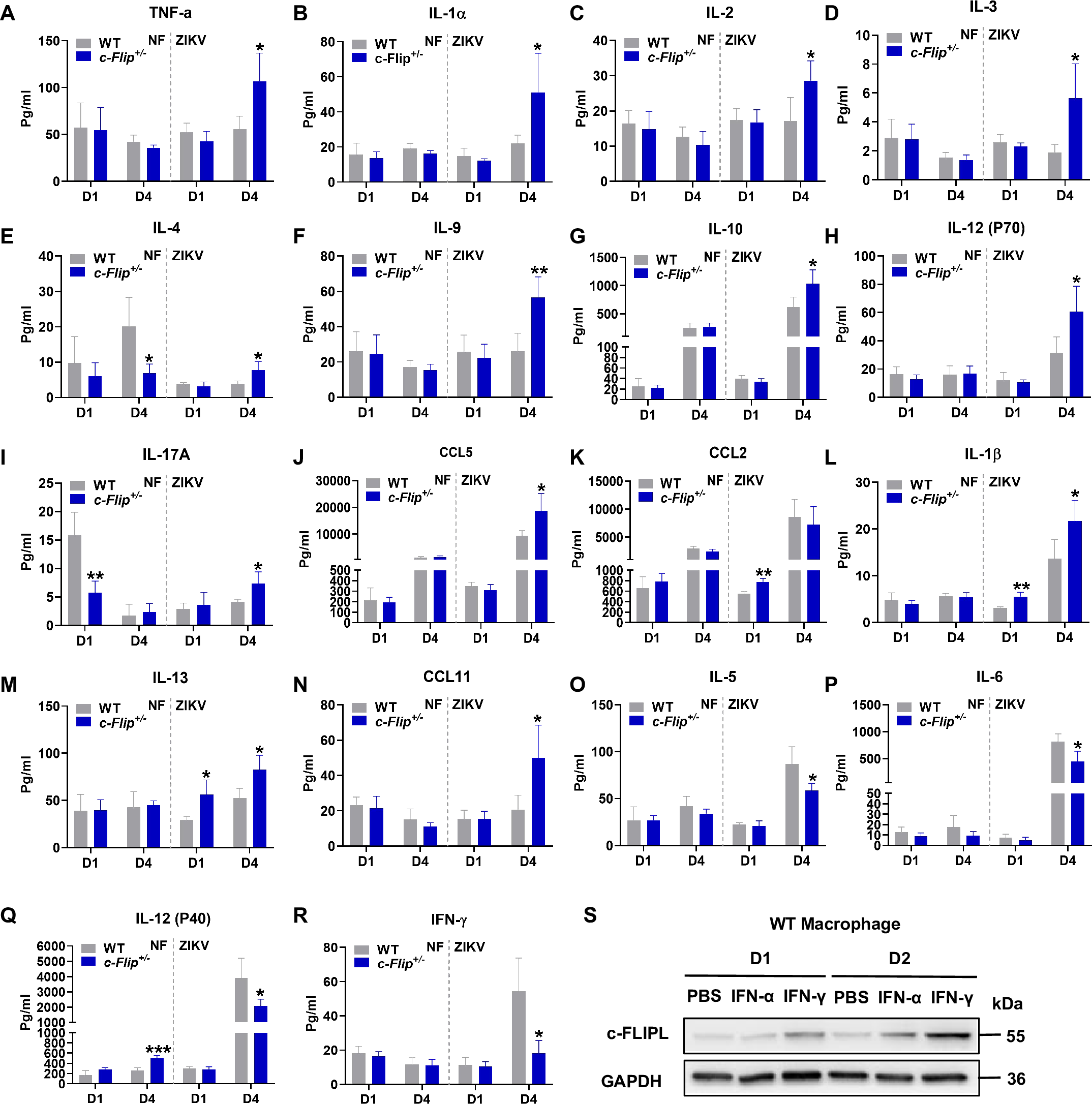

Supplement: S7 Fig — (A-R) Cytokine levels in the supernatant of ZIKV infected WT or c-Flip+/- macrophages (MOI = 1). Data are analyzed by unpaired Student’s t test and are presented as means ± SD. *P <0.05, **P < 0.01, ***P < 0.001, ****P < 0.0001 compared to control group. (S) WT macrophages were treated with PBS, IFN-α (20ng/ml) and IFN-γ (20ng/ml) respectively, and the expression level of c-FLIPL was measured by western blot. (TIF) [file ppat.1012408.s007.tif]

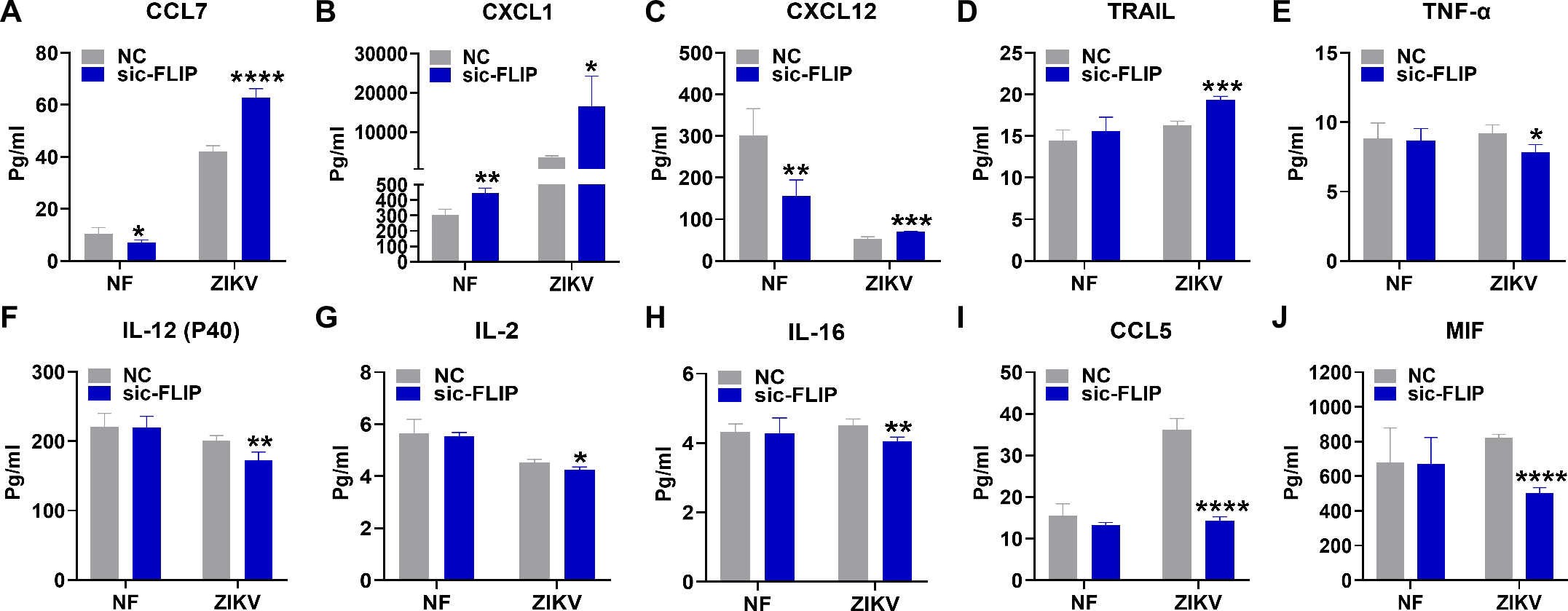

Supplement: S8 Fig — (A-J) HTR8 cells were infected with ZIKV at an MOI of 1 post sic-FLIP transfection for 24h. Cytokine levels in the supernatant of HTR8 cells were measured on D3 post-infection. Data are analyzed by unpaired Student’s t test and presented as means ± SD. *P <0.05, **P < 0.01, ***P < 0.001, ****P < 0.0001 compared to control group. (TIF) [file ppat.1012408.s008.tif]
