# Supplementary material for: c-FLIP facilitates ZIKV infection by mediating caspase-8/3-dependent apoptosis
Source: PLoS Pathog. 2024 Jul 22;20(7):e1012408. doi: 10.1371/journal.ppat.1012408 (PMC11293698; doi:10.1371/journal.ppat.1012408)
Supplement: S2 Table — (DOCX) [file ppat.1012408.s010.docx]

**S2 Table.** Primers for qPCR used in this study.

| Primer | Sequence 5’ to 3’ |
| --- | --- |
| Human GAPDH Forward | CTGACTTCAACAGCGACACC |
| Human GAPDH Reverse | TAGCCAAATTCGTTGTCATACC |
| ZIKV NS5 Forward | GGTCAGCGTCCTCTCTAATAAACG |
| ZIKV NS5 Reverse | GCACCCTAGTGTCCACTTTTTCC |
| Human c-FLIPL Forward | GCTGACCATCCCTGTACCTG |
| Human c-FLIPL Reverse | CAGGAGTGGGCGTTTTCTT |
| Human c-FLIPS Forward | TCTCCAAGCAGCAATCCAA |
| Human c-FLIPS Reverse | TCACATGGAACAATTTCCAAGAATTTT |
| Human c-FLIPR Forward | CAAGCAGCAATCCAAAAGAGTCT |
| Human c-FLIPR Reverse | TCATGCTGGGATTCCATATGTTT |
| Human caspase-3 Forward | TTAATAAAGGTATCCATGGAGAACACT |
| Human caspase-3 Reverse | TTAGTGATAAAAATAGAGTTCTTTTGTGAG |
| Human caspase-8 Forward | TGTCCTTCCTGAGGGAGCTGCT |
| Human caspase-8 Reverse | TGAGCCCTGCCTGGTGTCTGAA |
| Mouse GAPDH Forward | AGGTCGGTGTGAACGGATTTG |
| Mouse c-FLIPL Reverse | GTCTATTCTGTGGATGTTCTTCAGGC |
