# Supplementary material for: c-FLIP facilitates ZIKV infection by mediating caspase-8/3-dependent apoptosis
Source: PLoS Pathog. 2024 Jul 22;20(7):e1012408. doi: 10.1371/journal.ppat.1012408 (PMC11293698; doi:10.1371/journal.ppat.1012408)
Supplement: Attachment — Submitted filename: Response to Reviewers.docx [file ppat.1012408.s012.docx]

Dear editor,

Thank you for allowing us to revise our manuscript, PPATHOGENS-D-24-00041, for submission to PLOS Pathogens.

We sincerely appreciate the editor and reviewers' valuable feedback, which we have incorporated to enhance the quality of our manuscript. In response to the reviewers' comments, we have addressed each point individually and provided our detailed responses below. Additionally, we have thoroughly revised the previous draft, highlighting all changes in yellow for clarity.

Response major questions to Reviewer #1:

1.A major conclusion of the paper is that c-FLIP enhances ZIKV replication through caspase3/8 dependent mechanisms. The authors should perform an experiment with a caspase 3 and/or 8 specific inhibitor to support their conclusion as off target effects of caspase knock down cannot be excluded with teh present data.

Response: We sincerely appreciate your valuable comment. As shown in Supplementary Figure 5F-5I, we have included additional experiments investigating the effects of the caspase-8 inhibitor (Z-IETD-FMK) and caspase-3 inhibitor (Ac-DEVD-CMK TFA) on ZIKV replication. In the results section, we have incorporated additional descriptions in lines 416-420 (highlighted in yellow) as follows: “In addition, we treated HTR8 cells separately with a caspase-8 inhibitor (Z-IETD-FMK) and a caspase-3 inhibitor (Ac-DEVD-CMK TFA). Comparative analysis with the control group revealed a significant reduction in both ZIKV mRNA levels and titers on days 2 and 3 post-infection (S5F-5I Fig).”

2.Western blots for full length caspase 3 and 8 should be included in all appropriate figures.

Response: We appreciate the reviewer's feedback and have addressed the suggestions by supplementing the western blot experiments to show the expression levels of full-length caspase-3 and caspase-8. This required repeating the entire set of experiments, and the updated results are now presented in Figures 5D-5E, 6D, and 7C.

In Figure 5D-5E, no significant changes were observed in the protein levels of total caspase-8 and caspase-3 for each group. In the results section, we have incorporated additional descriptions in lines 327-329 (highlighted in yellow) as follows: “However, we did not observe any significant differences in the protein levels of cleaved caspase-8, total caspase-8, and total caspase-3 among the three groups.”

In Figure 6D, we observed that the protein levels of full-length caspase-8 and caspase-3 changed in the non-infected group and upon ZIKV infection. Thus, we have rephrased the descriptions in lines 361-371 (highlighted in yellow) as follows: “As depicted in Figure 6D, there was a reduction in total caspase-8 and caspase-3 protein levels on day 1 (lane 2 vs lane 1) and in full-length caspase-8, full-length caspase-3, and cleaved caspase-3 on day 4 (lane 6 vs lane 5) in *c-Flip^+/–^* macrophages compared to WT macrophages in the non-infected group. This indicates that c-FLIP may be involved in regulating the expression of caspase-8 and caspase-3 as well as the activation of caspase-3 in myeloid macrophages. Notably, ZIKV infection led to a noticeable increase in cleaved caspase-8 (Fig 6D, lane 3 vs lane 1, lane 7 vs lane 5) on days 1 and 4, and in cleaved caspase-3 (Fig 6D, lane 7 vs lane 5) on day 4 post-infection in WT macrophages. However, this activation was mostly diminished in the *c-Flip^+/–^* macrophages (Fig 6D, lane 4 vs lane 3, lane 8 vs lane 7).”

In Figure 7C, we observed the effect of c-FLIP on the protein levels of caspase-8 and caspase-3. Therefore, we have rephrased the corresponding description in lines 392-400 (highlighted in yellow) as follows: “As shown in Fig 7C, decreased levels of full-length and cleaved caspase-8/caspase-3 were observed in HTR8 cells transfected with sic-FLIP compared to the control, both in the non-infected group and with ZIKV infection, on day 1 and day 2. Based on the quantification, the ratio of cleaved caspase-8 and caspase-3 to total caspase-8 and caspase-3 is significantly lower in the c-FLIP knockdown group compared to the control group upon ZIKV infection while no significant was observed in non-infected group (S4A-4C Fig). This indicates reduced caspase-8 and caspase-3 activity with the reduction of c-FLIP expression upon ZIKV infection.”

3.Western blots for c-FLIP levels in different tissues should be included.

Response: We appreciate the reviewer's reminder. In Supplementary Figure 2C-2F, we have included additional data on the detection of c-FLIPL protein levels in the brain, uterus, and testis of mice. The rephrased sentence is indicated in lines 187-189 (highlighted in yellow) as follows: “The genotypes were verified by PCR analysis of genomic DNA (S2B Fig) and the reduced expression of c-FLIPL was confirmed at protein levels in spleen, brain, uterus, and testis (S2C-2F Fig).”

Response minor questions to reviewer #1:

4.Figure 1a. The authors claim a significant increase in c-FLIPL with ZIKV infection, but this is only moderately apparent at one timepoint, and the authors ignore that c-FLIPL increases in expression throughout the time course in the non-infected control. The authors should acknowledge and comment on this in their presentation of the results.

Response: We appreciate the reviewer's feedback. Indeed, we found that ZIKV induced a moderate increase in *c-FLIPL* mRNA at one timepoint. Therefore, we rephrased the sentence in lines 144-147 (highlighted in yellow) as follows: “Our analysis revealed a moderate increase in *c-FLIPL* mRNA levels on day 2 and a substantial increase of *c-FLIPS* replication on day 2, day 4, and day 6 (Fig 1A-1B) in ZIKV-infected HTR8 cells, as compared to the non-infected group.”

For the second concern, the review is absolutely right. Based on Figure 1A-1D, we have found that both c-FLIPL and c-FLIPS were increased throughout the time course in the non-infected control. We cannot figure out the reason based on our data right now, but we will investigate further in the future. Thus, we have rephrased the sentence in lines 153-157 (highlighted in yellow) as follows: “Interestingly, we observed an increase in the transcription (Fig 1A -1B) and protein (Fig 1C-1D) expression levels of c-FLIPL and c-FLIPS over time in the uninfected group, which we will investigate further. Nevertheless, these results indicate an augmentation of c-FLIPL and c-FLIPS expression in ZIKV-infected HTR8 cells at the indicated timepoints.”

5.Figure S1. The western blots are very low resolution and so difficult to interpret. However, it appears there is a major effect of the non-targeting control siRNA on teh expression of c-FLIPL between 24 and 48 hours. This should be quantified and reported.

Response: We appreciate the reviewer's feedback and have incorporated the suggestion. As shown in Supplementary Figure 1B and 1C, we conducted three replicates of the experiments and quantified the outcomes. We have rephrased the sentence as indicated in lines 157-161 (highlighted in yellow) as follows: “Next, we executed siRNA knockdown experiments, achieving a reduction of at least 50% in the mRNA (S1B Fig) levels of c-FLIPL, c-FLIPR, and c-FLIPS, as well as the protein levels of c-FLIPL and c-FLIPR (S1C-S1D Fig) in HTR8 cells followed by infection with the Asian-lineage strain (ZIKV-GZ01).”

6.Figure 1. The authors swap between days and hours for treatment in the different panels making comparison difficult. for example is D1 in panel A equivalent to 24 hours in panel C, if so there is a discord between the gene expression and protein levels that should be explained.

Response: We greatly appreciate your insightful feedback. In our manuscript, we've standardized the time notation across all figures, with day 1 representing 24 hours and day 2 representing 48 hours.

Regarding the observed discrepancies between gene expression and protein levels, we believe it is a reasonable outcome. RNA levels may not always directly correlate with protein levels due to various post-transcriptional processes, including mRNA stability, translation efficiency, and post-translational modifications.

7.Figure 2. There is a broad range in the degree of responses to ZIKV infection in different tissues. Does this reflect differential expression levels of c-FLIP (or caspase 3 or 8) in different tissues? Only western blot data is shown for spleen in fig S2, data for other organs should also be included for comparison.

Response: We sincerely appreciate your valuable feedback. In response, we have included the expression profiles of c-FLIPL in various tissues including brain, uterus, and testis in Supplementary Figure 2C-2F. Unfortunately, we were unable to compare these tissues on a single gel due to the strong c-FLIPL band in the spleen, which hindered the detection of c-FLIPL in the brain. We believe that the differential expression levels of c-FLIPL across different tissues may contribute to variations in viral replication. Additionally, we think that the tissue tropism for ZIKV is complex; for example, brain infection requires crossing the blood-brain barrier. Therefore, a deeper understanding requires more data, and we will study it further in the future.

8.Figure 5a-c. Apoptotic processes and negative regulation of apoptosis processes are highlighted, however, many more processes are also presented with much greater proportion of gene changes, with the highlighted changes quite far down the list. The authors should acknowledge this as roles of other important pathways cannot be excluded.

Response: The reviewer is correct. We indeed observed activation of other important biological processes, which could offer valuable insights for further exploration into the pathogenic mechanisms of ZIKV. Currently, we are studying the role of these processes in ZIKV infection, although our investigations are still in the initial stages. We genuinely appreciate your suggestion. Thus, we have rephrased the discussion in lines 330-334 (highlighted in yellow) as follows: “While our focus was primarily on caspase-8 and caspase-3 due to the known association with c-FLIP and the subsequent activation of effector caspase-3, we acknowledge the significance of other biological processes highlighted by the RNA-seq data. It's important not to overlook these processes in our investigation.”

9.Figure 5d and 5e. Western blot data for full length caspase 8 and caspase 3 should be included.

Response: We appreciate the reviewer's feedback and have addressed the suggestions by supplementing the western blot experiments to show the expression levels of full-length caspase-3 and caspase-8. This required repeating the entire set of experiments, and the updated results are now presented in Figure 5D-5E. In Figure 5D-5E, no significant changes were observed in the protein levels of total caspase-8 and caspase-3 for each group. In the results section, we have incorporated additional descriptions in lines 327-329 (highlighted in yellow) as follows: “However, we did not observe any significant differences in the protein levels of cleaved caspase-8, total caspase-8, and total caspase-3 among the three groups.”

10.Figure 6c. Western blot data for full length caspase 8 and caspase 3 should be included.

Response: We appreciate the reviewer's feedback and have addressed the suggestions by supplementing the western blot experiments to show the expression levels of full-length caspase-3 and caspase-8. This required repeating the entire set of experiments, and the updated results are now presented in Figure 6D. In Figure 6D, we observed that the protein levels of full-length caspase-8 and caspase-3 changed in the non-infected group and upon ZIKV infection. Thus, we have rephrased the descriptions in lines 361-371 (highlighted in yellow) as follows: “As depicted in Figure 6D, there was a reduction in total caspase-8 and caspase-3 protein levels on day 1 (lane 2 vs lane 1) and in full-length caspase-8, full-length caspase-3, and cleaved caspase-3 on day 4 (lane 6 vs lane 5) in *c-Flip^+/–^* macrophages compared to WT macrophages in the non-infected group. This indicates that c-FLIP may be involved in regulating the expression of caspase-8 and caspase-3 as well as the activation of caspase-3 in myeloid macrophages. Notably, ZIKV infection led to a noticeable increase in cleaved caspase-8 (Fig 6D, lane 3 vs lane 1, lane 7 vs lane 5) on days 1 and 4, and in cleaved caspase-3 (Fig 6D, lane 7 vs lane 5) on day 4 post-infection in WT macrophages. However, this activation was mostly diminished in the *c-Flip^+/–^* macrophages (Fig 6D, lane 4 vs lane 3, lane 8 vs lane 7).”

11.Figure 7C. Authors should include the western blot gels for total caspase 8 and caspase 3 in these experiments.

Response: We appreciate the reviewer's feedback and have addressed the suggestions by supplementing the western blot experiments to show the expression levels of full-length caspase-3 and caspase-8. This required repeating the entire set of experiments, and the updated results are now presented in Figure 7C. In Figure 7C, we observed the effect of c-FLIP on the protein levels of caspase-8 and caspase-3. Therefore, we have rephrased the corresponding description in lines 392-400 (highlighted in yellow) as follows: “As shown in Fig 7C, decreased levels of full-length and cleaved caspase-8/caspase-3 were observed in HTR8 cells transfected with sic-FLIP compared to the control, both in the non-infected group and with ZIKV infection, on day 1 and day 2. Based on the quantification, the ratio of cleaved caspase-8 and caspase-3 to total caspase-8 and caspase-3 is significantly lower in the c-FLIP knockdown group compared to the control group upon ZIKV infection while no significant was observed in non-infected group (S4A-4C Fig). This indicates reduced caspase-8 and caspase-3 activity with the reduction of c-FLIP expression upon ZIKV infection.”

12.The test should be carefully read to correct minor text errors.

Response: We sincerely appreciate the reviewer’s reminder. We found several minor errors in the manuscript and have rectified them. We have listed the following examples. Firstly, we have corrected the term “at an MOI of 1” to “at a MOI of 1” (highlighted in yellow) in line 168. Secondly, in line 184 of the text, we have revised the word “*c-Filp*" to "*c-Flip*” (highlighted in yellow). Additionally, we have modified the word “macrophage” to “macrophages” in line 382.

Response major questions to Reviewer #2:

1.Fig. 1A and Fig. 1B: normalisation for the y-axis is required. Assessing fold-change differences between infected and non-infected cells and between c-FLIPL and c-FLIPS is very difficult.

Response: We appreciate the reviewer's feedback and have incorporated the suggestion. We have performed fold changes conversion for the mRNA levels of c-FLIPL and c-FLIPS in Figure 1A and 1B. We observed a significant increase in c-FLIPL mRNA levels on day 2 (Fig 1A) and a substantial increase of c-FLIPS replication on day 2, day 4, and day 6 (Fig 1B) in ZIKV-infected HTR8 cells, as compared to the non-infected group. This finding remains consistent with our original results, and we have retained the same description.

2.Fig. 1C: it is indicated in the text that “these results indicate an augmentation of c-FLIPL and c-FLIPS expression in ZIKV-infected HTR8 cells”. However, this is very difficult to assess with Fig. 1C, and some quantification, at least in triplicate, is required.

Response: We appreciate the reviewer's feedback. As shown in Figure 1D, we have quantified the protein expression levels of c-FLIPL and c-FLIPS in HTR8 cells infected with ZIKV infection. This has been mentioned in lines 147-149 and line 173 of the manuscript (highlighted in yellow).

3. In S1A, why are the levels for c-FLIPL and c-FLIPS so different from Fig. 1A?

Response: We appreciate the reviewer's feedback. After carefully examining the data presented in the two figures, we did not identify any inappropriate analysis. However, we acknowledge that the transfection process may impact cellular status, which can, in turn, affect c-FLIP expression via this technique, and this influence may be unavoidable. As suggested, we modified the data presentation to show fold increase in Figure 1A and Supplementary Figure 1A, allowing for a clearer observation of the change trend. The results are consistent with what we previously observed.

4.In S1B, why are protein levels for c-FLIPL in non-treated cells lower at 48h compared to 24h? In Fig. 1C, these levels are increasing with time. In addition, protein quantification is required.

Response: The reviewer is correct. To verify this inconsistency, we quantified the three replicates of the experiments for Supplementary Figure 1B and presented the results in Supplementary Figure 1C. We observed that both c-FLIPL and c-FLIPS protein levels increased significantly on day 2 compared to day 1 in non-treated cells. Therefore, we replaced Supplementary Figure 1B with a new image as a representative of this trend. We have rephrased the descriptions in lines 157-161 (highlighted in yellow) as follows: “Next, we executed siRNA knockdown experiments, achieving a reduction of at least 50% in the mRNA (S1B Fig) levels of c-FLIPL, c-FLIPR, and c-FLIPS, as well as the protein levels of c-FLIPL and c-FLIPR (S1C-S1D Fig) in HTR8 cells followed by infection with the Asian-lineage strain (ZIKV-GZ01).”

For the second concern, the review is absolutely right. Based on Figure 1A-1D, we have found that both c-FLIPL and c-FLIPS were increased throughout the time course in the non-infected control. We cannot figure out the reason based on our data right now, but we will investigate further in the future. Thus, we have rephrased the sentence in lines 153-157 (highlighted in yellow) as follows: “Interestingly, we observed an increase in the transcription (Fig 1A -1B) and protein (Fig 1C-1D) expression levels of c-FLIPL and c-FLIPS over time in the uninfected group, which we will investigate further. Nevertheless, these results indicate an augmentation of c-FLIPL and c-FLIPS expression in ZIKV-infected HTR8 cells at the indicated timepoints.”

5.It is stated in the text that the homozygous c-FLIP^-/-^ genotype is lethal. How is the cell viability in silenced cells? Can this be assessed to discard the reduction in virus replication due to lower cell viability?

Response: We appreciate the reviewer's feedback and have addressed the suggestion. As depicted in Supplementary Figure 1D, we assessed cell viability following the knockdown of c-FLIP in HTR8 cells. We have incorporated additional descriptions in lines 151-163 (highlighted in yellow) as follows: “Notably, silencing c-FLIP expression in HTR8 cells using siRNA did not result in any significant effect on cell viability compared to transfection with negative control siRNA (S1E Fig).”

6.Assessing differences in c-FLIP expression in the different mouse tissues would be very interesting. It could explain why partial knock-down of c-FLIP in the spleen is more detrimental to virus replication than in the brain. Also, is this related to the different isoforms? How are the different isoforms expressed in the different tissues?

Response: We sincerely appreciate your valuable comment. We have added the protein levels of c-FLIPL in the brain, uterus, and testis in Supplementary Figure 2C-2F. As the reviewer pointed out, we found that c-FLIPL expression appears to be higher in the spleen, and the knockdown efficiency seems greater compared to the brain. In fact, we cannot even detect c-FLIPL expression in the brain when spleen and brain tissue lysates are run on the same gel due to the strong band from the spleen. This could be one reason why partial knockdown of c-FLIP in the spleen is more detrimental to virus replication than in the brain. Additionally, we observed that partial knockdown of c-FLIP in the uterus and testis is more detrimental to virus replication than in the brain, despite their expression levels being comparable to those in the brain. We speculate that c-FLIP might also play a complex role in ZIKV crossing the blood-brain barrier, and we will investigate this further.

Regarding the different isoforms of c-FLIP, we were initially very interested in whether the ratio of different isoforms could affect ZIKV infection and made multiple attempts to measure their expression in various mouse tissues. However, as stated in lines 150-152 (highlighted in yellow), c-FLIPR was found to be undetectable in the human cell line. Additionally, we were only able to detect c-FLIPL in the mouse tissues. Many studies have also indicated that their research only detected c-FLIPL in mouse (PMID: 19109151; 23424201; 2267567) or both c-FLIPL and c-FLIPS in human cell lines (PMID: 37988267, 35115486). Considering this is an interesting topic, we will investigate it further if we can develop an antibody in the future to detect the isoforms.

7.The information about partial demise and growth restriction in ZIKV-infected WT pregnant dams is very scarce in the text. More clarification is required to understand the presented results.

Response: We sincerely appreciate the reviewer's comments. We have rephrased descriptions in lines 233-238 (highlighted in yellow) as follows: “On E13.5, we observed that the fetuses from ZIKV-infected WT group in the uterus underwent fetal demise and subsequent absorption, leaving behind only placental remnants (Red arrows). While isolated cases of microcephaly were not observed in the ZIKV-infected WT group, pallor was evident (Green arrows). However, fetuses from ZIKV-infected *c-Flip^+/–^* group developed normally without miscarriage or absorption, and did not exhibit pallor (Fig 3B-3C).”

8.The list of enriched genes by RNA-Seq presented in Fig. 5A is required for each group to assess which genes have been included in each category.

Response: The reviewer is correct. We made a mistake in the description from line 301 to 302. We have incorporated correct descriptions in lines 312-314 (highlighted in yellow): “Gene Ontology (GO) enrichment analysis revealed the top 20 significantly enriched biological processes associated with differential expression of genes (DEGs).” Additionally, we listed the enriched differential expression of genes for each biological process in Figure 5A-5C into the excel forms which will be upload as S1 Table.

9.In Figs. 5D, 5E and 6C, several panels for other viral and cellular proteins are required to strengthen the results. First, a comparison with non-infected animals is needed. This also needs to include a blot for a viral protein. In addition, it is only possible to assess how much caspases 8 and 3 have been cleaved with the blots for the uncleaved caspases. This will also require quantification in multiple replicates.

Response: The reviewer is correct. Originally, our intention was to compare the differences between the fetal heads from the non-infected and ZIKV-infected groups to gain insight into the biological processes c-FLIP is involved in. However, due to the heterogeneity in our in vivo experiments, it has been challenging to draw clear conclusions. Even the wild-type (WT) fetal heads from WT dams or *c-Flip^-/-^* dams showed differences in multiple biological process. With six groups for each panel, the results were confusing. Additionally, when we injected the virus directly into the dams to mimic maternal transmission, the virus protein was barely detectable in the fetal head. Since we were primarily looking for preliminary clues in Figure 5D-5E, we focused on detailed mechanisms using mouse--derived macrophages. For Figure 6C, now modified as Figure 6D, we included non-infected controls, viral mRNA, and virus released in the supernatant (Fig 6A-6B). We believe these data sufficiently address the reviewer's concerns.

For the second concern, we have addressed it by repeating all the western blot experiments to include the full length of caspase-8 and caspase-3. We have attempted to summarize the ratio of cleaved caspase/total caspase for the related figures, such as Figure 7C listed in Supplementary Figure 4A-4C. However, the qualifications have several panels, making the comparison too complex for our intended point. While it's common practice to assess apoptosis by examining the cleaved forms of caspase-8 and caspase-3, as highlighted in several articles (PMID: 37494451, 37988267), it is also easier for us to present our results in this manner. We sincerely hope for the reviewer's understanding.

10.In Fig. 6, it is stated that “c-FLIP positively regulates caspase-8 and caspase-3 mediated apoptosis”; however, this is overstated without repeating these experiments with a positive control for apoptosis induction independently of virus infection.

Response: We sincerely appreciate your valuable comment and have incorporated the suggestion. As shown in Figure 6C, we have supplemented MG132 (an apoptosis inducer) (PMID: 21499287) as a positive control to observe whether c-FLIP in murine macrophages induces apoptosis by promoting caspase-8 and caspase-3 activation. We have incorporated additional descriptions in lines 358-359 (highlighted in yellow) as follows: “Firstly, we have confirmed that c-FLIP facilitates caspase-8 and caspase-3 activation upon apoptosis induced by MG132, an apoptosis inducer (Fig 6C).”

11.Fig. 6A and 6B show minimal differences between WT and c-FLIP-/- macrophages, probably due to poor infection. Is it possible to repeat these infections in more permissive cell lines where cFLIP can be silenced, or can this be repeated with a higher MOI?

Response: We appreciate the reviewer's feedback and have incorporated the suggestion. As shown in Figure 6A and 6B, we repeated the experiments using a higher titer of ZIKV (MOI=3). We observed a trend consistent with our previous findings, showing a significant reduction in both viral mRNA levels (Fig 6A) and viral titers (Fig 6B) in primary myeloid *c-Flip^+/–^* macrophages compared to WT group on both day 1 and day 4 post-infection. Additionally, macrophages exhibited higher mRNA levels and viral titers on day 4 at an MOI of 3 compared to an MOI of 1. Accordingly, we have supplemented the results in Figure 6A and Figure 6B. We have rephrased descriptions in lines 353-354 (highlighted in yellow) as follows: “To enhance permissiveness, the macrophages were pre-treated with MAR1-5A3 followed by ZIKV infection at a MOI of 1 or 3.”

12.In Fig. 7C, quantifying the uncleaved/cleaved caspase ratio is necessary to discern whether a lower amount of active caspase 8/3 is due to less apoptosis or lower mRNA levels.

Response: We appreciate the reviewer's feedback. We assume that the reviewer would like to know if the lower amount of active caspase 8/3 is due to the reduction of total caspase 8/3. As listed in Supplementary Figure 4A-4C, we have found that the cleaved/total caspase ratio is significantly lower with the knockdown of c-FLIP upon ZIKV infection while no significant was observed in non-infected group, indicating less active caspase with the reduction of c-FLIP expression upon ZIKV infection. we have rephrased the corresponding description in lines 392-400 (highlighted in yellow) as follows: “As shown in Fig 7C, decreased levels of full-length and cleaved caspase-8/caspase-3 were observed in HTR8 cells transfected with sic-FLIP compared to the control, both in the non-infected group and with ZIKV infection, on day 1 and day 2. Based on the quantification, the ratio of cleaved caspase-8 and caspase-3 to total caspase-8 and caspase-3 is significantly lower in the c-FLIP knockdown group compared to the control group upon ZIKV infection while no significant was observed in non-infected group (S4A-4C Fig). This indicates reduced caspase-8 and caspase-3 activity with the reduction of c-FLIP expression upon ZIKV infection.”

13.In Fig. 7D and E, it needs to be better explained how apoptosis has been measured by flow cytometry and immunofluorescence. What is etoposide and annexin-V measuring?

Response: We appreciate the reviewer's feedback. Recombinant human Annexin V labeled with the fluorescent probe FITC can be used to detect phosphatidylserine exposed on the cell membrane surface during apoptosis. Phosphatidylserine is primarily distributed on the inner leaflet of the cell membrane. In the early stages of apoptosis, phosphatidylserine translocates to the outer leaflet of the cell membrane. Annexin V binds to phosphatidylserine exposed on the cell surface, allowing for the simple and direct detection of this important feature of apoptosis using flow cytometry or fluorescence microscopy. Etoposide is an apoptosis inducer, triggering GSK-3-mediated mitochondrial-dependent apoptotic signals (PMID: 27473919). We have rephrased the sentence as indicated in lines 409-414 (highlighted in yellow) as follows: “Subsequently, we induced apoptosis in HTR8 cells using etoposide. We then utilized Annexin V labeled with FITC to measure the release of phosphatidylserine to the cell surface induced by apoptosis. Finally, the FITC signal was detected using immunofluorescence assay (IFA) (Fig 7D) and flow cytometry (Fig 7E). The apoptosis is significantly attenuated in HTR8 cells transfected with sic-FLIP (Fig 7D right panel and Figure7E).”

14.In S4A and S4B, according to the Western blot, the knockdown efficiency of the different caspases seems to be less than 50%. Can this be quantified and provide mRNA levels of these transcripts?

Response: We sincerely appreciate your valuable comment and have incorporated the suggestion. We have supplemented experiments demonstrating the downregulation of caspase-8 and caspase-3 mRNA expression levels using siRNA In Supplementary Figure 5B and 5C. In addition, we have quantified the protein expression levels of caspase-8 and caspase-3. These results demonstrated that knockdown efficiency of the different caspases more than 50% on day 2. We have rephrased descriptions in lines 414-415 (highlighted in yellow) as follows: “We ensured a knockdown efficiency of at least 50% (S5B-S5E Fig) followed by ZIKV infection.”

15.In Fig. 7G and I, the viral titres in HTR8 non-silenced cells are very low compared to those in Fig. 1F. How can this discrepancy be assessed?

Response: The reviewer is correct. Upon careful review of the data, we discovered that two different students conducted the experiments separately, with an interval of two years and in different locations due to our relocation to a new city. Uncertainty regarding which step in the experiment may have encountered issues prompted us to repeat the experiments three times under current conditions with a third student. Upon conducting these repetitions, we observed that the replication level of ZIKV and the viral titers in HTR8 cells with c-FLIP knocked down were not as high as initially observed. Consequently, we have updated Figure 1E-1F to clarify.

16.In Fig. 8, indicate in the text how the cytokines and chemokines levels have been assessed.

Response: We appreciate the reviewer's feedback. We have supplemented the detection steps of cytokines in the materials and methods section. We have incorporated additional descriptions in lines 666-675 (highlighted in yellow) as follows: “Add 20μL sample (including diluted mouse plasma, serum and HTR8 cells supernatant) and 35μL beads to each well, then incubate at 37°C for 1 hour. Following the incubation, wash the beads by adding 400μL wash buffer. Subsequently, add 20μL of detection antibody and incubate at 37°C for 35 minutes, followed by another wash of the magnetic beads. Next, add 50 μL Bio-Plex SA-PE (Streptavidin-Phycoerythrin) and incubate for 15 minutes, and wash the beads once more. Utilize the Bio-Plex 200 System to read the data, and Bio-Plex ManagerTM Software presentsdata as median fluorescence intensity (lMFl) as well as concentration (pg/ml). The concentration of the cytokines and chemokines bound to each bead correlates with the reported signal MFI. Finally, employ the Bio-Plex Data ProTM software for data analysis.”

17.Fig. 8N and S5R: the potential positive regulatory role of c-FLIP in type II IFN can be further investigated by adding IFN lambda to cells.

Response: Thanks to the reviewer’s suggestion, we assumed that the reviewer want to know the role of type II IFN (IFN-γ rather than IFN lambda) in regulating c-FLIP. We have included additional experiments examining the effects of type I (IFN-α) and type II interferons (IFN-γ) on the expression levels of c-FLIPL protein in WT mouse macrophages. We observed that c-FLIPL expression was up-regulated following stimulation with both IFN-α and IFN-γ. Therefore, we have added these results to line 460-464 (highlighted in yellow): “As previous studies indicate that interferon promotes c-FLIP expression (PMID: 23230268), we wondered if type I IFN and type II IFN affect c-FLIP expression in macrophages. Interestingly, an elevation in the protein expression of c-FLIPL in WT macrophages following treatment with both IFN-α and IFN-γ was evident (S6S Fig), indicating a potential positive regulatory role of c-FLIP for both type I and type II interferons.”

18.The mechanism of how c-FLIP influences the extrinsic apoptosis pathway upon infection must be further discussed regarding the extensive published literature. For instance, how c-FLIP interacts with pro-caspase 8. Also, it will be interesting to know how c-FLIP is upregulated during infection. Is this only at the transcriptional level or also at the post-translational level?

Response: We appreciate the reviewer's valuable comment. For the mechanism of how c-FLIP influences the extrinsic apoptosis pathway upon infection, we have added the discussion in lines 493-510 (highlighted in yellow) as follows: “During the death receptor signal transduction process, a complex known as the death-inducing signaling complex (DISC) is formed upon the stimulation of a death signal. Both c-FLIP and caspase-8 participate in the formation of DISC. Upon stimulation, DISC is formed, initiating the pro-apoptotic caspase cascade and ultimately leading to cell death (PMID: 22309778). The isoforms of c-FLIP can bind to caspase-8 through its N-terminal death-effector domains (DEDs), and their concentrations subtly regulate caspase-8 activation. c-FLIPL contains two DEDs and a caspase-like domain (CLD). When c-FLIPL acts at low or moderate concentrations, it promotes the formation of heterodimers between c-FLIPL and caspase-8, thereby facilitating the activation of caspase-8 at the DISC. However, at higher concentrations of c-FLIPL, it competitively binds to the DEDs of caspase-8, inhibiting its activation (PMID: 34973957). As the short isoforms, both c-FLIPS and c-FLIPR lacks the CLD domain but retains the DEDs. The DEDs of c-FLIPS are followed by 20 amino acids crucial for its ubiquitination and subsequent targeting for proteasomal degradation. In contrast, c-FLIPR contains two DEDs but lacks the additional carboxy (C)-terminal amino acids present in c-FLIPS (PMID: 23070002). The differential expression of c-FLIPR and c-FLIPS was observed in several cell lines. Furthermore, c-FLIPR and c-FLIPS exhibited comparable biochemical characteristics in their recruitment to the DISC (PMID: 15701649).”

19.c-FLIP has an influence not only on apoptosis but also on necroptosis and autophagy. Are there differences in the intrinsic apoptosis pathway, necroptosis or autophagy in c-FLIP silenced cells upon ZIKV infection?

Response: We appreciate the reviewer's feedback and have incorporated the suggestion. As shown in Supplementary Figure 5A, we conducted additional experiments to assess the expression levels of the necroptosis marker pMLKL protein and the autophagy marker LC3B protein after knocking down c-FLIP. In the results section, we have added the following description in line 400-409 (highlighted in yellow) as follows: “Due to the role of c-FLIP in necroptosis and autophagy, we also investigated whether these processes are involved in the regulation of ZIKV infection. As shown in Supplementary Figure 5A, we observed a decrease in the expression levels of the necroptosis-associated protein pMLKL on day 2 in non-infected HTR8 cells transfected with si-cFLIP, while ZIKV infection showed no significant effect on the total MLKL and pMLKL protein levels. This suggests that ZIKV does not involve necroptosis in HTR8 cells through the c-FLIP pathway. Notably, there was no significant change in the expression of the autophagy-related protein LC3B. These results indicate that c-FLIP-related necroptosis and autophagy are not involved during ZIKV infection of HTR8 cells.”

20.The interplay of the different c-FLIP isoforms during ZIKV infection and apoptosis activation should be further discussed.

Response: We appreciate the reviewer's feedback. It is a limitation for us to determine the isoforms of c-FLIP during ZIKV infection since our technique cannot measure c-FLIPS in mouse and c-FLIPR in human cell lines. We have added a discussion a in lines 510-513 (highlighted in yellow) as follows: “Although we are greatly interested in studying the roles of different isoforms during ZIKV infection, we were unable to detect all the isoforms and determine their accurate concentrations. We will investigate this further once we obtain an antibody that can detect all the isoforms of c-FLIP.”

21.Have the authors planned to repeat the most relevant experiments with an isolate of the African lineage? This will strengthen the observed results or clarify differences in virulence between lineages.

Response: We appreciate the reviewer’s suggestions. We originally intended to use the African strains, as they may be more destructive and lead to fetal demise (PMID: 29903417). However, we were unable to obtain this strain for multiple reasons. We would like to repeat the most relevant experiments with an isolate of the African lineage if possible.

Response minor questions to reviewer #2

22. What is the difference between c-FLIPS and c-FLIPR? This needs to be clarified in the text.

Response: We thanks the reviewer’s reminder. we have included a sentence in the introduction section to delineate the difference between c-FLIPS and c-FLIPR in lines 493-510 (highlighted in yellow) as follows: “During the death receptor signal transduction process, a complex known as the death-inducing signaling complex (DISC) is formed upon the stimulation of a death signal. Both c-FLIP and caspase-8 participate in the formation of DISC. Upon stimulation, DISC is formed, initiating the pro-apoptotic caspase cascade and ultimately leading to cell death (PMID: 22309778). The isoforms of c-FLIP can bind to caspase-8 through its N-terminal death-effector domains (DEDs), and their concentrations subtly regulate caspase-8 activation. c-FLIPL contains two DEDs and a caspase-like domain (CLD). When c-FLIPL acts at low or moderate concentrations, it promotes the formation of heterodimers between c-FLIPL and caspase-8, thereby facilitating the activation of caspase-8 at the DISC. However, at higher concentrations of c-FLIPL, it competitively binds to the DEDs of caspase-8, inhibiting its activation (PMID: 34973957). As the short isoforms, both c-FLIPS and c-FLIPR lacks the CLD domain but retains the DEDs. The DEDs of c-FLIPS are followed by 20 amino acids crucial for its ubiquitination and subsequent targeting for proteasomal degradation. In contrast, c-FLIPR contains two DEDs but lacks the additional carboxy (C)-terminal amino acids present in c-FLIPS (PMID: 23070002). The differential expression of c-FLIPR and c-FLIPS was observed in several cell lines. Furthermore, c-FLIPR and c-FLIPS exhibited comparable biochemical characteristics in their recruitment to the DISC (PMID: 15701649).”

23. Change the c-FILP typo for c-FLIP.

Response: We appreciate the reviewer's reminder. We have revised this mistake.

24. Rephrase “higher ZIKV replication in their sperms”; what has been assessed is the viral load in the spermatic liquid.

Response: We appreciate the reviewer's feedback and have incorporated the suggestion. In the results section, we have rephrased descriptions in lines 204-206 (highlighted in yellow) as follows: “On day 7 post-infection, we observed higher ZIKV replication in the testis, epididymis, and their spermatic liquid (Fig 2G).”

25. In Fig. 3A and Fig. 4A, the representation of a pregnant dam at days E5.5 and E6.5 would be easier to understand than the performed experiment.

Response: We appreciate the reviewer's reminder. In Figure 3A and Figure 4A, we have replaced the images representing E5.5 and E6.5 mice with pregnant dam.

26. Include a table with the sequences of the primers used.

Response: We appreciate the reviewer's feedback and have incorporated the suggestion. We have supplemented the information in Supplementary Table 1.

Response major questions to Reviewer #3:

1.Some critical controls are missing. For instance, the authors conclude that c-FLIP promotes zika virus infection by modulating caspase-3/-8 apoptosis. While the authors show decreased levels of cleaved caspase-3 and -8, the authors should also include the levels of pro-caspase-3 and -8 levels (Figures 5D, E, 6C, and 7C) to support this claim.

Response: We appreciate the reviewer's feedback and have addressed the suggestions by supplementing the western blot experiments to show the expression levels of full-length caspase-3 and caspase-8. This required repeating the entire set of experiments, and the updated results are now presented in Figures 5D-5E, 6D, and 7C.

In Figure 5D-5E, no significant changes were observed in the protein levels of total caspase-8 and caspase-3 for each group. In the results section, we have incorporated additional descriptions in lines 327-329 (highlighted in yellow) as follows: “However, we did not observe any significant differences in the protein levels of cleaved caspase-8, total caspase-8, and total caspase-3 among the three groups.”

In Figure 6D, we observed that the protein levels of full-length caspase-8 and caspase-3 changed in the non-infected group and upon ZIKV infection. Thus, we have rephrased the descriptions in lines 361-371 (highlighted in yellow) as follows: “As depicted in Figure 6D, there was a reduction in total caspase-8 and caspase-3 protein levels on day 1 (lane 2 vs lane 1) and in full-length caspase-8, full-length caspase-3, and cleaved caspase-3 on day 4 (lane 6 vs lane 5) in *c-Flip^+/–^* macrophages compared to WT macrophages in the non-infected group. This indicates that c-FLIP may be involved in regulating the expression of caspase-8 and caspase-3 as well as the activation of caspase-3 in myeloid macrophages. Notably, ZIKV infection led to a noticeable increase in cleaved caspase-8 (Fig 6D, lane 3 vs lane 1, lane 7 vs lane 5) on days 1 and 4, and in cleaved caspase-3 (Fig 6D, lane 7 vs lane 5) on day 4 post-infection in WT macrophages. However, this activation was mostly diminished in the *c-Flip^+/–^* macrophages (Fig 6D, lane 4 vs lane 3, lane 8 vs lane 7).”

In Figure 7C, we observed the effect of c-FLIP on the protein levels of caspase-8 and caspase-3. Therefore, we have rephrased the corresponding description in lines 392-400 (highlighted in yellow) as follows: “As shown in Fig 7C, decreased levels of full-length and cleaved caspase-8/caspase-3 were observed in HTR8 cells transfected with sic-FLIP compared to the control, both in the non-infected group and with ZIKV infection, on day 1 and day 2. Based on the quantification, the ratio of cleaved caspase-8 and caspase-3 to total caspase-8 and caspase-3 is significantly lower in the c-FLIP knockdown group compared to the control group upon ZIKV infection while no significant was observed in non-infected group (S4A-4C Fig). This indicates reduced caspase-8 and caspase-3 activity with the reduction of c-FLIP expression upon ZIKV infection.”

2.In Figure 5 A-C, the authors conduct gene ontology analyses of genes that are differentially expressed as a result of Zika infection both in WT and in c-Flip+/- cells. In my opinion, it will be more informative to compare genes that are differently expressed as a result of c-FLIP expression (comparing WT versus c-Flip+/- cells) to understand the impact of c-Flip knockdown in the transcriptome and in response to Zika infection.

Response: The reviewer’s comment is accurate. We indeed compared the DEGs between WT and *c-Flip^+/-^* fetal heads. The results yielded informative data, and we intend to draft them into another manuscript. Upon comparing the non-infected group with the infected group, we believe that the data from the infected group are more likely to reflect the role of c-FLIP during ZIKV infection.

3.In Figure 1B, the authors compare the expression of c-FLIP in non-infected versus Zika-infected cells over 6 days in the human cell line HTR8. It is surprising that the levels of c-FLIP increase over time by 3-fold even in the non-infected conditions. One explanation is that c-FLIP is regulated by autophagy (and activated due to media starvation), which in turn can regulate interferon stimulated genes (ISGs). It will be important to show that c-FLIP is not an ISG, given that the in vivo and ex vivo results were conducted in the presence of an interferon signalling neutralizing antibody.

Response: The reviewer’s comment is accurate. We did overlook the increasing c-FLIP levels throughout the time course in the non-infected group. Therefore, we have pointed out that in lines 153-157 (highlighted in yellow) as follows: “Interestingly, we observed an increase in the transcription (Fig 1A -1B) and protein (Fig 1C-1D) expression levels of c-FLIPL and c-FLIPS over time in the uninfected group, which we will investigate further. Nevertheless, these results indicate an augmentation of c-FLIPL and c-FLIPS expression in ZIKV-infected HTR8 cells at the indicated timepoints.”

As the reviewer suggested, we conducted the additional experiments examining the effects of type I and type II interferons on the expression levels of c-FLIPL protein in WT mouse macrophages. We observed that c-FLIPL expression was up-regulated following stimulation with both IFN-α and IFN-γ in Supplementary Figure 6S, indicating c-FLIP is an ISG. we have added these results to line 460-464 (highlighted in yellow): “As previous studies indicate that interferon promotes c-FLIP expression (PMID: 23230268), we wondered if type I IFN and type II IFN affect c-FLIP expression in macrophages. Interestingly, an elevation in the protein expression of c-FLIPL in WT macrophages following treatment with both IFN-α and IFN-γ was evident (S6S Fig), indicating a potential positive regulatory role of c-FLIP for both type I and type II interferons.”

Response minor questions to Reviewer #3:

4. It has been difficult to assess some of the main figures, given the low resolution of the figures that were provided to me.

Response: We appreciate the reviewer's reminder. We have adjusted the font and resolution of the figures.

5. Please revise the statistical tests used throughout the manuscript as Student t-test analyses are used even when comparing multiple groups.

Response: We appreciate the reviewer's feedback and have incorporated the suggestion. In Figure 3D-3F and Figure 4D-4G, we changed the statistical method from Student t-test to Turkey test of ANOVA analysis. In Figure 3D-3E and Figure 4D-4G, the statistical difference observed remains consistent with previous findings except Figure 3F. In Figure 3F, we observed that the ZIKV mRNA level in WT fetal heads is slightly higher compared to *c-Flip^+/–^* fetal head from the same mother, however, this difference did not reach statistical significance as previous analyses. In the results section, we have revised descriptions in lines 247-251 (highlighted in yellow) as follows: “ZIKV replication in the WT fetal head was increased compared to the *c-Flip^+/–^* fetal heads. Intriguingly, a trend of higher ZIKV replication levels in WT fetal heads compared to *c-Flip^+/–^* fetal heads from the same dam was noted, although this difference did not reach statistical significance (Fig 3F).”

6. p-values are missing in figure 5A-C.

Response: We appreciate the reviewer's reminder. As shown in Figure 5A-5C, we reanalyzed the data and represented p-values using different colors to indicate their significance levels.

7. Please provide quantification and scale bar in Figure 7D.

Response: We appreciate the reviewer's feedback and have incorporated the suggestion. In Figure 7D, we added a scale bar (left panel) and quantified the effect of c-FLIP knockdown on apoptosis in HTR8 cells (right panel). After transfection with sic-FLIP, we observed a notable decrease in cellular fluorescence (green) in HTR8 cells upon quantification. We have rephrased the sentence as indicated in lines 409-414 (highlighted in yellow) as follows: “Subsequently, we induced apoptosis in HTR8 cells using etoposide. We then utilized Annexin V labeled with FITC to measure the release of phosphatidylserine to the cell surface induced by apoptosis. Finally, the FITC signal was detected using immunofluorescence assay (IFA) (Fig 7D) and flow cytometry (Fig 7E). The apoptosis is significantly attenuated in HTR8 cells transfected with sic-FLIP (Fig 7D right panel and Figure7E).”
