# Supplementary material for: c-FLIP facilitates ZIKV infection by mediating caspase-8/3-dependent apoptosis
Source: PLoS Pathog. 2024 Jul 22;20(7):e1012408. doi: 10.1371/journal.ppat.1012408 (PMC11293698; doi:10.1371/journal.ppat.1012408)
Supplement: Attachment — Submitted filename: Response to Reviewers.docx [file ppat.1012408.s013.docx]

Dear editor,

We sincerely appreciate the valuable feedback from the editor and reviewers, which has greatly helped us improve the quality of our manuscript. In response to Reviewer 2, we have addressed each point individually and provided detailed responses below. Additionally, we have thoroughly revised the previous draft and highlighted all changes in yellow for clarity.

Response minor questions to Reviewer #2:

1. Line 146: What does c-FLIPS replication mean in this context?

Response: We sincerely appreciate the reviewer's reminder. The expression regarding c-FLIPS replication was not appropriate. Therefore, we have rephrased the descriptions in lines 144-147 (highlighted in yellow) as follows: “Our analysis revealed a moderate increase in *c-FLIPL* mRNA levels on day 2 and a substantial increase of *c-FLIPS* mRNA levels on day 2, day 4, and day 6 (Fig 1A-1B) in ZIKV-infected HTR8 cells, as compared to the non-infected group.”

2. Fig 1D: c-FLIPL is missing in the y-axis.

Response: We appreciate the reviewer's reminder. We have reviewed and updated the image in Figure 1D.

3. Indicate the titres plot in Fig 1F and discuss their fold-reduction.

Response: We appreciate the reviewer's feedback. In Figure 1F, we have used PFU/ml as the unit of viral titers instead of log_10_ PFU/ml to provide a clearer evaluation of the differences between groups. Additionally, we performed a fold change analysis on the data in Supplementary Figure 1F, and the results have been rephrased accordingly in lines 163-166 (highlighted in yellow) as follows: “As depicted in Fig 1E-1F and S1F Fig, the viral loads in ZIKV-infected HTR8 cells demonstrated a 20% decrease on day 2 and a 40% decrease on day 3 post-infection compared to control siRNA-treated cells, as assessed by quantitative polymerase chain reaction (qPCR) and plaque assay.”

4. According to Figure 2, lines 227-228 need to be rephrased as they are not true.

Response: The reviewer is right. According to the results of Figure 2, we mistakenly described “the decreased ZIKV replication in reproductive organs of *c-Flip^+/-^* mice” as “the increased ZIKV replication in reproductive organs of *c-Flip^+/-^* mice” in line 227. Thus, we have rephrased the descriptions in lines 227-228 (highlighted in yellow) as follows: “Considering the decreased ZIKV replication in reproductive organs of *c-Flip^+/-^* mice, we subsequently explored the impact of c-FLIP knockdown on CZS.”

5. Line 333: remove the abbreviation of “it’s”

Response: We appreciate the reviewer's feedback. We have rephrased the descriptions in lines 333-334 (highlighted in yellow) as follows: “It is important not to overlook these processes in our investigation.”

6. Although differences are significant in Figs 6A and 6B, it would be interesting to discuss further the fold-change differences, which, according to the graph, are minimal.

Response: We appreciate the reviewer's feedback. In Figure 6B, we have used PFU/ml as the unit of viral titers instead of log_10_ PFU/ml to provide a clearer evaluation of the differences between groups. As shown in Supplementary Figures 4A and 5B, we performed a fold change analysis on the data in Figures 6A and 6B, and the results have been rephrased accordingly in lines 354-361 (highlighted in yellow) as follows: “The viral mRNA levels in *c-Flip^+/-^* macrophages demonstrated a 40% decrease both on day 1 and day 4 post-infection (MOI=1), and a 40% decrease on day 1 and 50% decrease on day 4 post-infection (MOI=3) compared to WT group (Figs 6A and S4A). The viral titers in *c-Flip^+/-^* macrophages demonstrated a 90% decrease on day 1 and a 70% decrease day 4 post-infection (MOI=1), and a 50% decrease on day 1 and 60% decrease on day 4 post-infection (MOI=3) compared to WT group (Figs 6B and S4B). These results indicate c-FLIP promotes ZIKV infection in myeloid cells.”

7. Apart from cleaved caspase 3, in Fig 6D, it is very difficult to assess caspase activation without proper quantification. Full-size blots in the Supplementary Information will be required to determine this.

Response: The reviewer is right. As we have quantified the expression of key proteins in Figure 1D in Supplementary Figures 4C-4E, we observed that the ratio of cleaved caspase-8 and caspase-3 to total caspase-8 and caspase-3 is significantly lower in the *c-Flip^+/-^* macrophages compared to the control group upon ZIKV infection on day 4 rather than both on day 1 and day 4. Therefore, we have rephrased the descriptions in lines 370-375 (highlighted in yellow) as follows: “Notably, ZIKV infection led to a noticeable increase in cleaved caspase-8 (Fig 6D, lane 7 vs lane 5) on day 4 (S4C-S4D Fig), and in cleaved caspase-3 (Fig 6D, lane 7 vs lane 5) on day 4 (S4E Fig) post-infection in WT macrophages. However, this activation was mostly diminished in the *c-Flip^+/-^* macrophages (Fig 6D, lane 8 vs lane 7) on day 4 (S4C-S4E Fig) post-infection.”

8. Fig 7D is very difficult to assess. Images with better contrast will be required to show annexin V-FITC.

Response: We appreciate the reviewer's feedback. We have reviewed and updated the image in Figure 7D. Since it is just one representative picture and difficult to assess in isolation, we have quantified the cell fluorescence in three different fields of view. The results are presented in the right panel of Figure 7D, showing that the knockdown of c-FLIP caused a 70% decrease in green fluorescence.
